# Development and optimization of pearl millet waste biocomposite ceiling tiles: a waste management approach

**DOI:** 10.1038/s41598-025-08351-1

**Published:** 2025-07-01

**Authors:** Khalid Hussain Ansari, Srikanta Routroy, Rahul Samyal, Shivasheesh Kaushik

**Affiliations:** Department of Mechanical Engineering, Birla Institute of Technology & Science, Pilani, Rajasthan 333031 India

**Keywords:** Agricultural waste management, Biocomposite, Taguchi optimization method, Sustainable thermal insulation, Pearl millet, Mechanical engineering, Sustainability

## Abstract

The present study aims to develop and optimize sustainable biocomposite ceiling tiles by partially replacing gypsum with pearl millet waste, combined with Waste Wheat Flour (WWF) and waste paper. It aims to provide a sustainable alternative to conventional gypsum-based product, maintaining thermal insulation and flexural strength. Taguchi method was employed for experimental design, by varying key parameters such as gypsum, Pearl Millet Seed Covering (PMSC), and ceiling tile thickness. The signal-to-noise (S/N) ratio was used to identify the optimal composition and Analysis of Variance (ANOVA) with regression analysis determines the significance of each factor on thermal conductivity and flexural strength. The optimized composition for minimum thermal conductivity (0.065 W/m·K) was identified as 45% PMSC and 10% gypsum at 12 mm thickness. For maximum flexural strength (1.24 MPa), the optimal mix was 55% PMSC and 30% gypsum at 14 mm thickness. The finding underscore the critical influence of gypsum and tile thickness in enhancing material performance. The developed regression models exhibited predictive R^2^ value of 96.90% for thermal conductivity and 94.44% for flexural strength and an error margin below 3%, confirming the robustness of the approach. This study is original and introduces pearl millet waste biocomposites as eco-friendly alternatives to gypsum ceiling tiles. These tiles hold practical implications in ceiling, partition walls and decorative finishes across various geographic regions, contributing significantly to environmental sustainability and energy-efficient building solutions.

**Graphical abstract:**

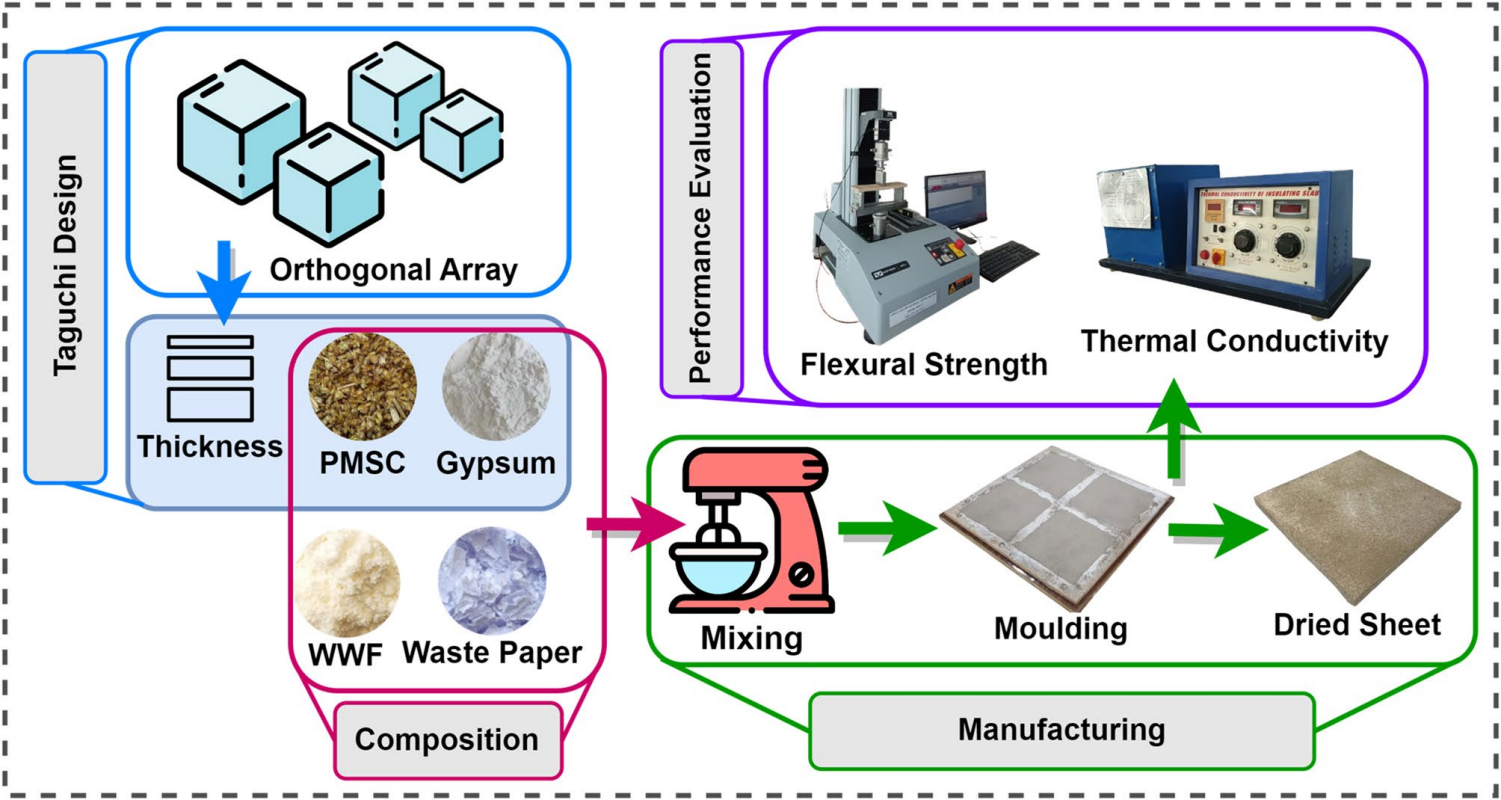

## Introduction

The building sector consumes nearly one third of the energy consumption globally. This makes it a critical sector of interest focusing on energy efficiency and sustainable development^1,2^. A significant portion of this energy is used for heating and cooling facilities to maintain indoor comfort under different weather conditions^3^. The combined emissions of buildings sector contribute substantially to climate change and environmental degradation. Energy consumption can be reduced by incorporating insulation to minimize heat transfer, thereby reducing need for artificial climate control, promoting sustainable building practices^4^. Effective insulation helps in maintaining stable indoor temperature, reducing the heating and cooling equipment loads^5,6^.

Gypsum ceiling tiles are widely used in building sector for several benefits, including aesthetic appearance and thermal insulation. Generally, such tiles have a thermal conductivity between 0.121 and 0.205 W/mK, making them effective indoor comfort materials^7^. While gypsum is a common choice for ceiling tiles, it has several limitations. It is derived from non-renewable sources and is non-biodegradable in nature. The extraction and processing of gypsum contribute to landscape degradation and intensive energy consumption^8^. Additionally, gypsum ceiling tiles have relatively low impact resistance^9^. Such tiles poses disposal challenge, as it releases hazardous hydrogen sulfide in landfills, contaminating recycling and health risks^10^. These limitations underscore the need for sustainable alternatives to gypsum ceiling tiles.

Globally, a large amount of agricultural waste is generated every year, reaching to approximately 140 billion metric tons^11,12^. Common disposal methods such as open burning and landfilling negatively impacts air, soil and water quality^13,14^. In particular, the burning of agricultural waste significantly contributes to climate change by releasing carbon and particulate matter, thereby increasing greenhouse gas concentrations^15,16^. Among various crop residues, pearl millet waste is commonly subjected to open burning due to the lack of sustainable disposal alternatives, further exacerbating environmental degradation. The total amount of crop residue (C_R_) generated estimated based on residue to crop ratio (N). The dry matter to crop residue ratio (D) was the factor that was used to calculate the dry matter from residue generation. The total dry matter generated was calculated by multiplying the crop production (P) by the residue to crop ratio and the dry matter to crop residue ratio (Eq. 1)^17,18^. According to government estimates, the average pearl millet production between 2020 and 2024 was around 10.71 million tonnes^19^. Using a residue to crop ratio (N) of 1.2 and a dry matter to residue ratio (D) of 0.9, the total amount of crop residue (C_R_) was estimated to be approximately 11.56 million tonnes^20–22^.

1 $$\:{\text{C}}_{\text{R}}=\text{P}\:.\:\text{N}\:.\:\text{D}.$$

Transforming agricultural waste into value-added products presents an effective solution to minimize the environmental damage associated with these practices. Moreover, it reduces the dependence on gypsum, a key raw material in the production of ceiling tiles^23^. Since agricultural waste is typically abundant and easily accessible, it can offers a cost-effective alternative that can partially replace gypsum, thereby lowering material expenses. The increasing demand for sustainable materials for indoor applications stems from the push toward energy efficiency and environmental sustainability. Natural fibers and their composites, offer excellent thermal performance and have lower environmental impacts^24^. These materials align with circular economy principles by reducing resource depletion and promoting waste recovery, contributing to eco-friendly construction practices^25^.

Several studies shown in Table 1 have explored agricultural waste to develop composites for indoor applications. Agricultural waste-based composites offer notable thermal insulation and mechanical properties, making them ideal for energy-efficient indoor applications. Studies highlight materials such as rice husk, coir, sugarcane bagasse, and wheat straw as key contributors to reducing thermal conductivity, with values as low as 0.047 W/mK^26–28^. Developed composites including hybrid blends of groundnut shells and coir or fiber-reinforced gypsum further optimize insulation performance^29^. By leveraging these sustainable materials, buildings can achieve better temperature regulation, reduced energy consumption and environmental benefits.

**Table 1:** Flexural and thermal properties of agricultural waste composites.

Agricultural waste	Flexural strength	Thermal insulation properties
Rice husk and coir fibers^26^	The study reports increase in flexural strength up to a 187% (5.6 MPa) compared to pure gypsum	Composites resulted decrease in thermal conductivity compared to pure gypsum, 0.075 W/mK
Coconut coir and sugarcane bagasse^27^	Flexural Strength increases with an increase in fiber content. 29.7 MPa and 39.9 MPa for 20% of sugarcane bagasse and coconut coir	Thermal conductivity decreases with an increase in fiber content. 0.342 W/mK and 0.303 W/mK for 20% of sugarcane bagasse and coconut coir
Wheat straw and corn husk^28^	The flexural strength reported, reaching about 0.80 MPa	The thermal conductivity values for the composites were found to be 0.047 W/mK
Groundnut Shell and Coir^29^	Hybrid composites reports increase in flexural strength, exhibited highest flexural strength of 3.6 MPa	The lowest thermal conductivity (0.066 W/mK) was observed in the composite with a balanced mix of waste
Rice Husk, Vine Pruning, Cork, Prickly Pear^30^	Inclusion of rice husk positively influences the flexural strength, ranged from 0.08 MPa to 0.28 MPa	Rice husk contributes to the low thermal conductivity ranges from 0.0495 W/mK to 0.0917 W/mK
Wheat straw and rice husk^32^	Composites reports increase in flexural strength, 1 MPa as per standards, except for the 10% wheat straw level, recorded 0.81 MPa	Composites leads to improved thermal insulation properties. 29% lower thermal conductivity was found (0.127 W/mK)
Wool and Coir Fibers^32^	Fiber-reinforced gypsum can be increased up to 90% when using an optimal fiber content of 30%	The study reports effective thermal conductivity for the hybrid composites range from 0.250 to 0.276 W/mK
Groundnut Shell and Rice Husk^33^	The flexural strength of the composites was reported to range from 27.2 to 37.6 MPa, depending on the composition	The thermal conductivity of the composites found range from 0.156 to 0.270 W/mK
Sugarcane Bagasse^34^	Composites demonstrated 63% higher flexural strength compared to commercially available gypsum-based ceiling tiles	Composites exhibited good thermal stability, maintaining their integrity up to 250 °C
Rice husk and seashells^35^	The highest recorded flexural strength was 8.14 MPa	Thermal conductivity of the ceramic boards recorded relatively low, 0.102 W/mK to 0.153
Laterite-based bricks with pearl millet waste^36,37^	Mechanical strength was found to be lower as per increased porosity and reduced material density	Thermal conductivity of the bricks reported 1.4 W/mK for pure laterite blocks and 0.29 W/mK for laterite blocks with millet waste

Several studies have demonstrated the potential of natural fibers in enhancing the performance of composite materials, particularly in lightweight and sustainable applications, as summarized in Table 2. Methodology including Taguchi Design of Experiments (DOE) and hybrid optimization techniques has been a common approach across these studies, enabling precise optimization of various material properties. DOE is a statistical technique used to plan, conduct, and analyze experiments efficiently. It helps in understanding the effects of multiple variables simultaneously and optimizing process performance^38^. It is particularly useful in optimizing the performance of construction materials such as ceiling tiles by systematically analyzing and improving their properties^39^.

Natural fibers such as jute, hemp, flax, sisal, banana, bamboo, abaca, and kenaf have been widely explored as reinforcements. Combining such fibers as reinforcements improves strength-to-weight ratios and contribute to sustainable practice. The application of composites, such as sisal-glass fiber or abaca-basalt fiber reinforcements, has resulted in enhanced mechanical performance and lightweight properties, suitable for automotive, construction, and engineering industries^40,41^. Further, fibers such as Chambira and areca fruit husk have shown potential as cost-effective alternatives with reasonable mechanical properties, positioning them as viable substitutes for synthetic fibers^42,43^. Similarly, bamboo fiber-reinforced composites have demonstrated excellent insulating properties and low density, making them ideal for engineering and construction materials^44^. Composites development from banana fiber has also been highlighted for their high strength, stiffness, and lightweight characteristics, essential for automotive and civil applications^45^. Collectively, these studies emphasize the growing importance of natural fiber-reinforced composites as sustainable solutions for engineering and construction materials, aligning with the global shift towards eco-friendly innovation. In parallel, studies using millet waste in masonry applications have also reported promising results. Laterite-based bricks reinforced with pearl millet waste, finds thermal conductivity was significantly reduced from 1.4 W/mK in pure laterite to 0.29 W/mK in bricks with millet waste. Mechanical strength decreased due to increased porosity and reduced material density^36,37^.

**Table 2:** Optimized agricultural waste composites using Taguchi method.

Natural fiber	Composite type	Methodology	Advantages	Application
Sisal fibers^40^	Hybrid composites (sisal and glass fibers)	Taguchi optimization technique	Improved mechanical performance with weight reduction.	Structures components aimed at reducing weight and carbon emissions.
Abaca and Basalt^41^	Hybrid Abaca-Basalt Fiber Reinforced Epoxy	L9 Orthogonal Array (Taguchi Method) and ANOVA	Improved strength-to-weight ratio compared to synthetic fibers.	Engineering applications requiring lightweight and strong materials.
Areca Fruit Husk Fiber^42^	Polymer composite reinforced natural fibers	Taguchi DOE and single response analysis	Improved strength as reinforcement and mechanical properties.	Potential substitute for synthetic fibers in various applications.
Chambira (Astrocaryum Chambira)^43^	Hybrid epoxy matrix reinforced with natural fibers	Taguchi method with Response Surface Methodology	Lightweight with reasonable mechanical properties.	Automotive industry and Civil construction.
Bamboo fiber^44^	Bamboo fiber reinforced unsaturated polyester composites	Hybrid Taguchi - Grey Relational Analysis Method	Low density leading to weight reduction and Good insulating properties.	Engineering materials, automotive components and construction materials.
Banana fiber^45^	Polyester-banana fiber composite	Taguchi method (using L9 orthogonal array)	High strength, stiffness and lightweight.	Automotive components and Construction materials.
Jute, hemp, flax, and sisal^46^	Combinations of natural fibers with polymers	Taguchi DOE	Improved mechanical properties and lightweight.	Automotive components and construction materials.
Jute fiber and Kenaf fiber^46^	Epoxy composites reinforced with kenaf and jute fibers	Taguchi design with Response Surface Methodology	Improved mechanical properties.	Automotive industry.

Despite extensive research on agricultural waste and natural fiber-reinforced composites, significant gaps remain in the exploration of less conventional bio-residues. Previous studies have demonstrated the effectiveness of rice husk, sugarcane bagasse, wheat straw and natural fibers such as jute, hemp, and sisal in enhancing thermal insulation and mechanical properties of construction materials. However, the potential of pearl millet waste has remained largely unexamined. The development of biocomposites incorporating waste as a functional material has not been reported in prior literature. Moreover, there is a notable absence of optimization studies using the Taguchi framework in the context of such waste-based biocomposites, remaining mechanical and thermal behavior underexplored.

The novelty lies in utilizing pearl millet waste in ceiling tiles in non-load-bearing construction element and evaluate its combined thermal and mechanical performance through Taguchi optimization framework. This dual focus on sustainability and functional efficiency, supported by statistical optimization, addresses a key gap in the literature and positions pearl millet waste as a viable alternative to gypsum in eco-friendly building applications such as ceiling, partition walls and decorative finishes across various geographic regions. Moreover, by diverting pearl millet waste to value-added product it addresses environmental challenge of open burning thereby promoting circular economy principles and sustainable waste management.

In this context, the present study addresses these research gaps by using Pearl Millet Seed Coverings (PMSC) as agricultural waste in the formulation of biocomposite ceiling tiles, wherein gypsum is partially replaced by waste and combined with waste wheat flour (WWF) and waste paper. The Taguchi method is employed to systematically optimize material properties through a limited number of experimental trials, focusing on gypsum content, waste proportion and tile thickness. Flexural properties used to analyze the mechanical strength, followed by thermal insulation performance through thermal conductivity measurements.

## Methodology

Various Design of Experiment (DOE) techniques, coupled with optimization methods, help derive optimal combinations of process variables. These techniques identify influential parameters, their interactions, and their individual effects on output variables^48^. Optimization involves selecting the best solution among different alternatives to maximize or minimize an objective function^49^. Taguchi method allows systematic and efficient investigation of multiple parameter with a minimal number of experiments, which in turn lowers the time and cost involved in the experimental process^50–52^. The Method was applied in this study to optimize the composition of biocomposites by partial replacing gypsum with pearl millet waste, focusing on thermal conductivity and flexural strength.

### Selection of parameters and levels

Using an L9 orthogonal array, three critical parameters were considered, as shown in Table 3. PMSC varied at 35%, 45%, and 55% levels, gypsum adjusted to 30%, 20%, and 10%, and ceiling tile thickness set at **0.12 mm, 0.13 mm, and 0.14 mm**. The selection of gypsum, waste, and tile thickness as control parameters was informed by pilot studies conducted prior to the formal optimization phase. Each parameter was assigned levels based on their experimental significance. The number of levels refers to the distinct values or settings assigned to each parameter under investigation. The number of levels can vary depending on the specific problem and experimental requirements. While the Taguchi method can accommodate various levels of factors, it is common to use two to three levels for each factor to keep the experiment size manageable, while still obtaining reliable and efficient results^53,54^.

**Table 3:** Taguchi design factors and Levels.

Parameter	Level 1	Level 2	Level 3
Gypsum (%)	30	20	10
PMSC (%)	35	45	55
Thickness (mm)	**0.12**	**0.13**	**0.14**

### Orthogonal array

Orthogonal arrays are matrices used in the Taguchi method to systematically arrange the levels of various factors in an experiment^55^. Common orthogonal arrays include L4, L8, L16, L32 for two-level factors, and L9, L27 for three-level factors^56^. An L9 orthogonal array was selected, enabling the evaluation of three parameters or factors at three levels across nine compositions, illustrated in Table 4. All experiments were conducted under controlled laboratory conditions to ensure consistency.

**Table 4:** L9 orthogonal array layout.

Composition	Selected orthogonal array and levels			Orthogonal array layout for experiment		
	Gypsum	PMSC	Thickness	Gypsum (%)	PMSC (%)	Thickness (mm)
1	1	1	1	30	35	12
2	1	2	2	30	45	13
3	1	3	3	30	55	14
4	2	1	2	20	35	13
5	2	2	3	20	45	14
6	2	3	1	20	55	12
7	3	1	3	10	35	14
8	3	2	1	10	45	12
9	3	3	2	10	55	13

### Biocomposite preparation

**Fig. 1:**
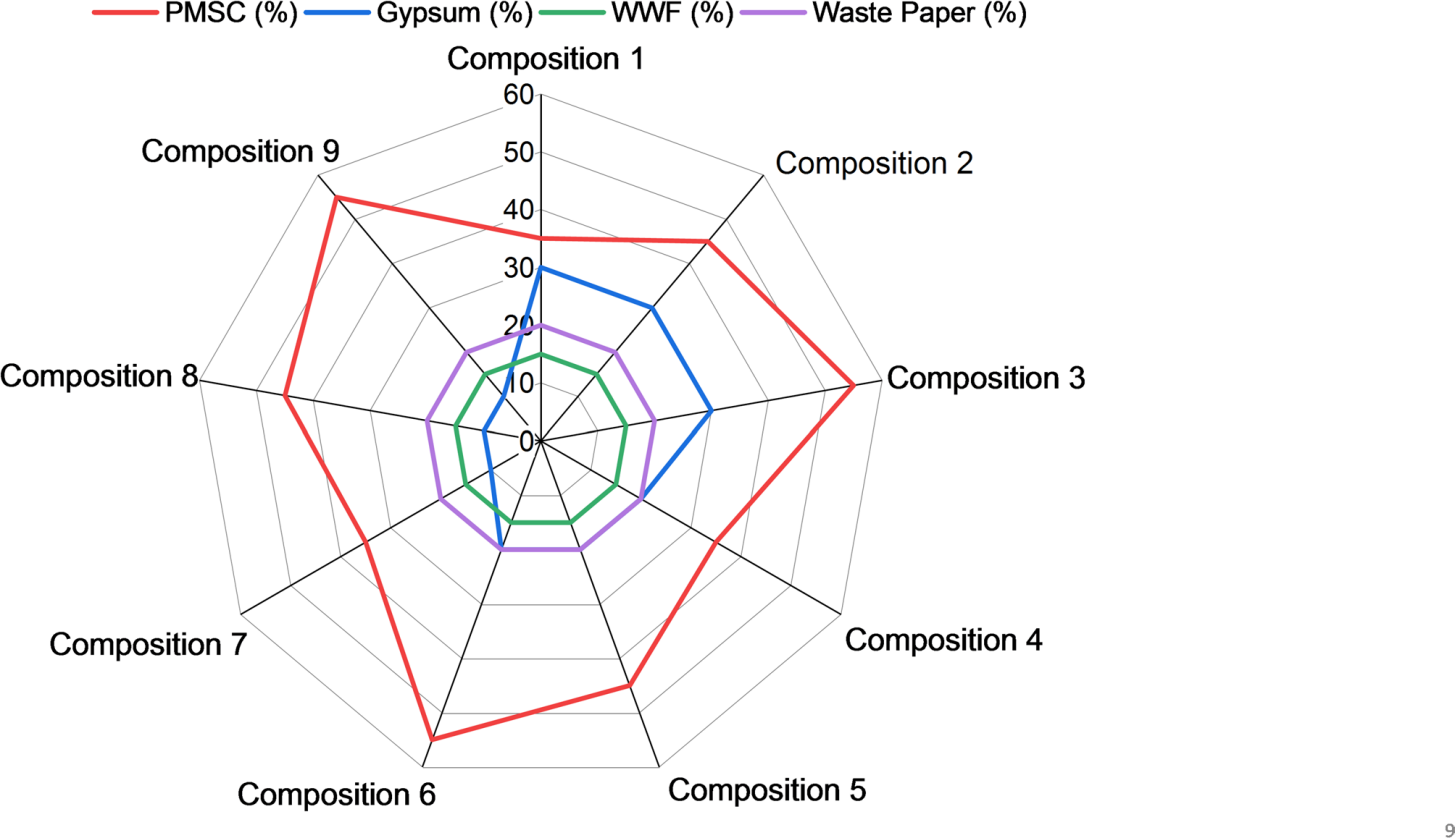
Design mix proportions.

Pearl millet waste used as a reinforcing material, collected from agricultural fields, cleaned to remove impurities and grinded into fine particles. Waste paper was incorporated as an additional fiber reinforcement. It was soaked in water for 10–12 h at a paper-to-water ratio of 1:4 (by weight) to soften the fibers and facilitate pulp formation. The inclusion of paper pulp fibers enhances the mechanical properties. Specifically, the cellulose fibers in paper waste contribute to increased strength and reduced brittleness of the composites (Balti et al., 2023). The soaked paper was further grinded to convert into pulp to prepare combined paste with WWF. WWF was sourced from a local flour mill and employed as a natural binder. It was prepared into paste form. The paste was prepared by mixing WWF and water in a 1:10 ratio by weight. The binding ability of wheat gluten arises from its unique composition of glutenin and gliadin proteins, which allow it to make it an effective binder for diverse applications^57–60^. In the second stage, the prepared combined paste of WWF and paper pulp mixed in a mechanical mixture with waste and gypsum. Gypsum serves as the primary matrix, providing a stable framework to bind all components (Barbero-Barrera et al., 2019; Murillo et al., 2024). The proposed mixture comprises 35–55% PMSC and 10–30% gypsum while keeping waste paper and WWF constant to 20% and 15%, respectively. The Design mix proportions in a dry state (% by wt.) are shown in Fig. 1.

**Fig. 2:**
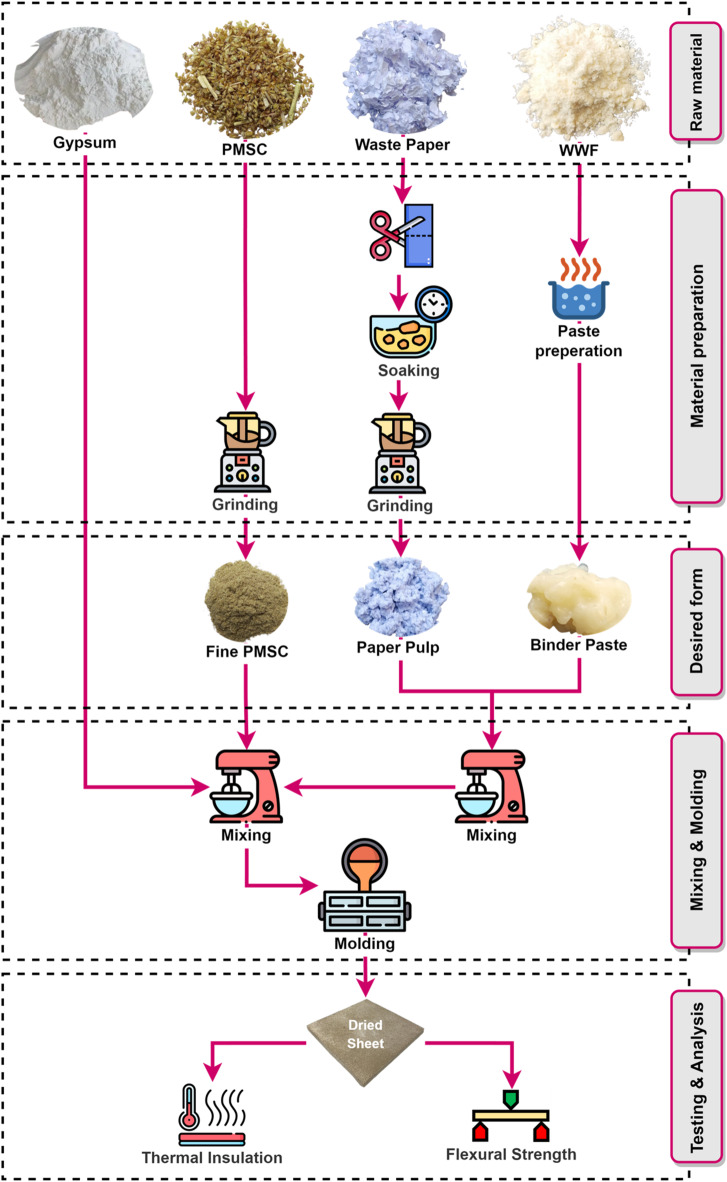
Flowchart of experimental method.

The prepared dough was placed into a mold with dimensions of 210 × 210 mm^2^ for molding. A thin layer of gypsum powder, approximately 0.5 mm thick, was applied to the outer surface of the molded tiles to enhance surface protection, improve aesthetics and increase resistance to impact and wear. Following molding, the tiles were subjected to drying and curing processes. Figure 2 presents the methodological framework.

### Testing procedures

#### Thermal conductivity test

**Fig. 3:**
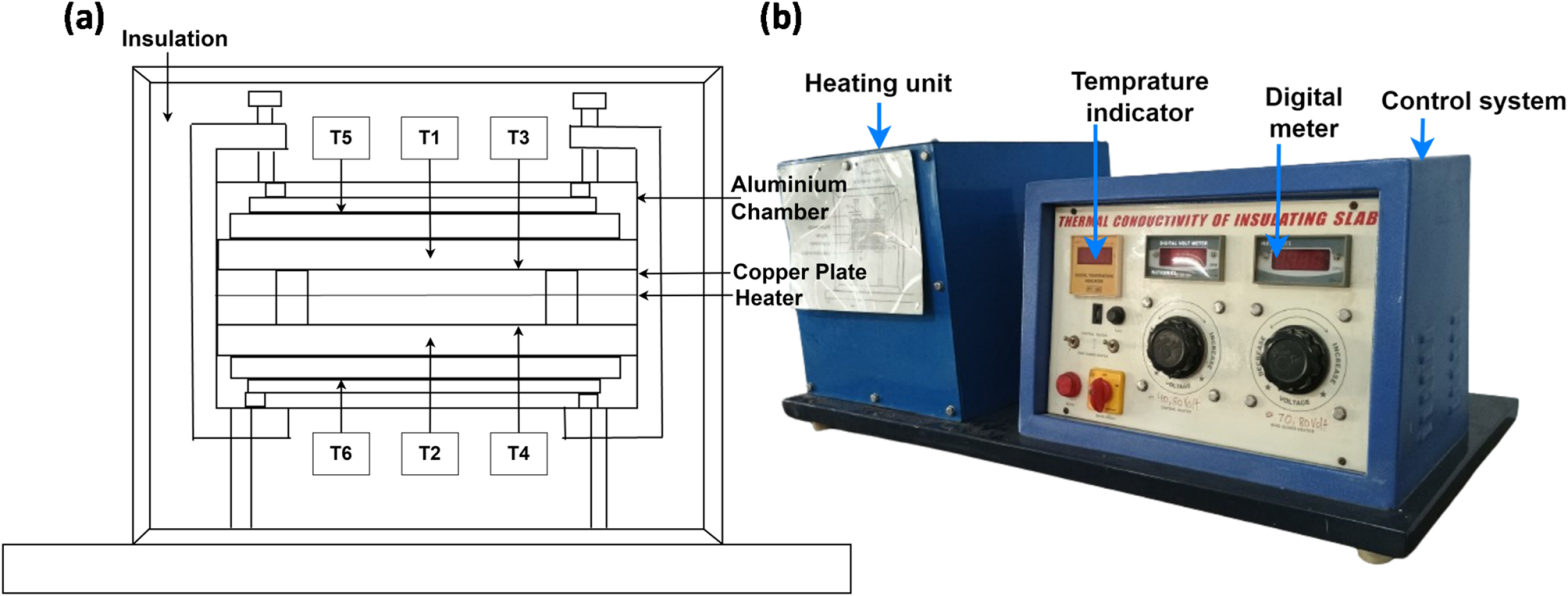
Schematic diagram (**a**) Actual setup (**b**) of thermal conductivity apparatus.

The guarded hot plate method, a widely recognized and precise technique for measuring the thermal conductivity of materials. It was employed in accordance with standard ASTM C177, 19^61^, within the temperature range of 60 °C. Figure 3a,b illustrate the schematic diagram and the actual setup of the guarded hot plate apparatus. The specimens were prepared by cutting the developed dried tile into standardized dimensions. The apparatus comprises a hot and cold plate with the test specimen sandwiched between them. The hot plate maintained at a higher temperature than the cold plate, resulting in a temperature gradient across the specimen. The temperatures at the hot plate surface (T_1_ and T_3_) and the cold plate surface (T_2_ and T_4_) were recorded to calculate the temperature gradient $\:{\Delta\:}T$ across the test specimen using expression in Eq. (2).

2 $$\:\varDelta\:T=\frac{{T}_{1}+{T}_{3}}{2\:}-\frac{{T}_{2}+{T}_{4}}{2\:}$$

Additionally, the temperatures at the guard plate (T_5_ and T_6_) were monitored to ensure uniform heat distribution and to minimize lateral heat losses, thereby maintaining a one-dimensional heat flow ($\:{T}_{5}{\approx\:T}_{6}$), ensuring minimal lateral heat dissipation. A steady-state temperature gradient was established between the hot and cold plates. Once steady-state condition was achieved, the temperatures at the hot, cold, and guard plates were recorded. The heat flux (*Q*) passing through the specimen was determined based on the applied voltage (*V*) and current (*I*) using Eq. (3).

3 $$\:Q=VI.$$

The thermal conductivity (*K*_*cond*_) was then calculated in *W/mK*, using Eq. (4). Where *d* specimen thickness (*m*), *A* is the surface areas (*m*^2^) and $\:{\Delta\:}T$ is the temperature difference in (K).

4 $$\:{K}_{cond}=\frac{Q\cdot\:d}{A\cdot\:{\Delta\:}T}.$$

#### Flexural strength test

**Fig. 4:**
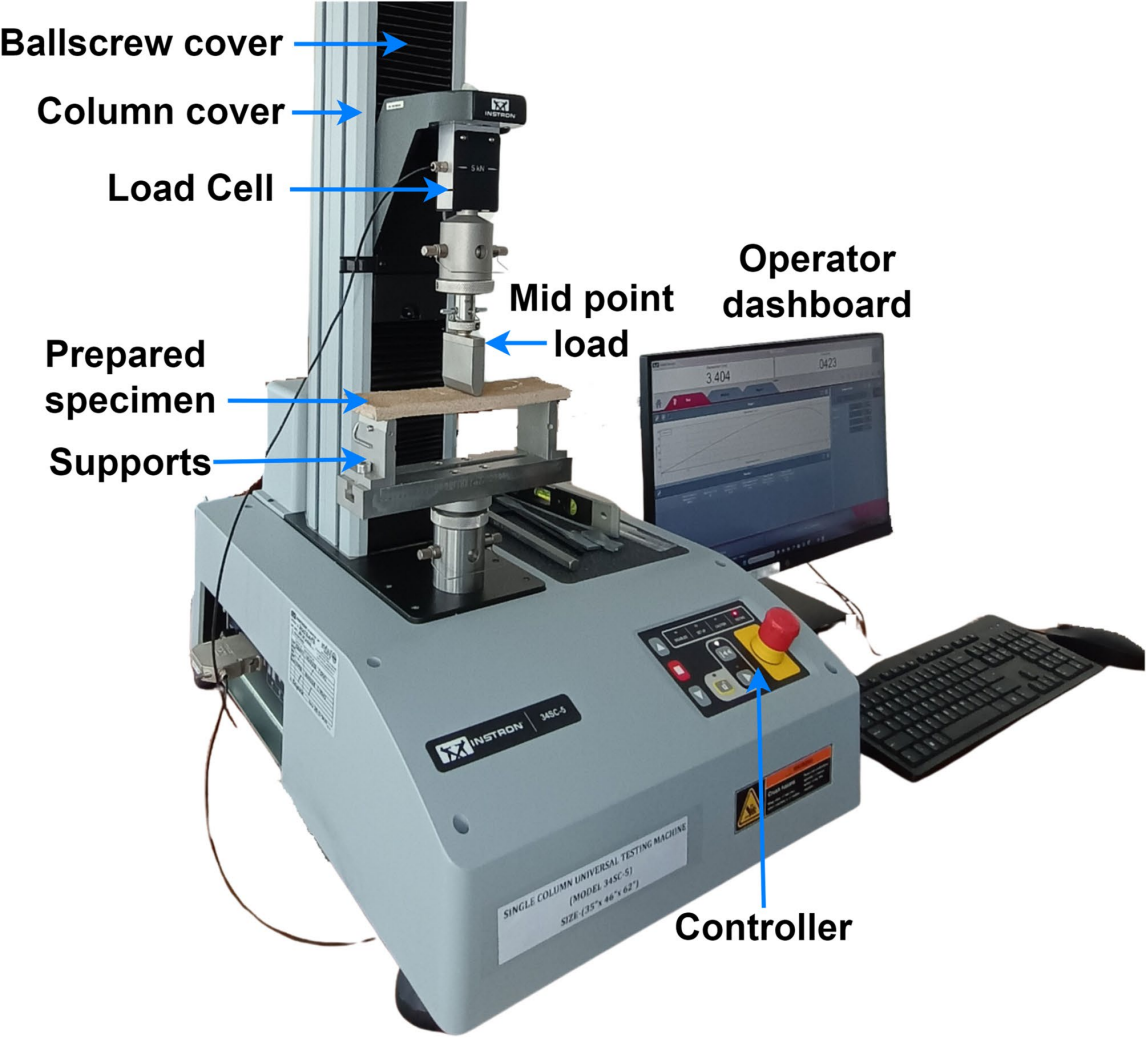
Universal testing machine setup for flexural strength.

Flexural strength refers to the ability of material to resist deformation under bending or flexural loads. This property is critical for determining the material resistance to fracture or deform under bending stresses, thereby ensuring its structural strength^62,63^. In the present study, the flexural strength was evaluated using the three-point bending test, the most common method for this purpose. The specimen was supported at both ends and the load was applied at the midpoint until the specimen fractures. The specimens were loaded to Instron 34SC-5, a single-column Universal Testing Machine (UTM) equipped with a 5 kN load cell. The testing was conducted in accordance with the standard ASTM C473^64–67^. The load was applied at a strain rate of 1 mm/minute. The setup, featuring the Universal Testing Machine and specimen, is depicted in Fig. 4.

### Data analysis

#### Signal-to-noise (S/N) ratio

Taguchi method uses Signal-to-Noise (S/N) ratio to quantify the quality of the applied characteristics^68,69^. The term signal represents the desirable value (mean) of the output characteristic, while noise denotes the undesirable variation (standard deviation) within the output. A higher S/N ratio signifies that the desired output is substantially greater than the random effects caused by noise factors. To optimize process parameters and minimize variability, Taguchi method employs objective functions tailored to the experimental goals, including Nominal-the-Best, Larger-the-Better and Smaller-the-Better^70,71^. In this study, achieving lower thermal conductivity and higher flexural strength was the primary objective. Consequently, Smaller-the-Better and Larger-the-Better S/N ratio characteristic was adopted for thermal conductivity and flexural strength, shown in Eqs. (5) and (6), respectively. Additionally, rank and delta values were used to determine the most influential parameters. The delta values represent the difference between the highest and lowest S/N ratios for each parameter, providing further insights into their significance.

5 $$\:S/N=-10\cdot\:{\text{l}\text{o}\text{g}}_{10}\left(\frac{1}{n}{\sum\:}_{i=1}^{n}\:{y}_{i}^{2}\right)$$

6 $$\:S/N=-10\cdot\:{\text{l}\text{o}\text{g}}_{10}\left(\frac{1}{n}{\sum\:}_{i=1}^{n}\:\frac{1}{{y}_{i}^{2}}\right).$$

#### Analysis of variance (ANOVA)

ANOVA is used to determine the significance of individual factors and their contributions to the overall response^72,73^. It offers a measure of confidence by analyzing data variance, computing sums of squares, variances, and relative contributions of each significant input parameter^74–76^. F-ratio measures the contribution of each factor or interaction to the variance of the response. A higher F-ratio indicates a more significant factor. P-value tests the statistical significance of each factor and interaction. A p-value ≤ 0.10 indicates significance at the 90% confidence level, while a p-value < 0.05 indicates significance at the 95% confidence level^77,78^. The results were analyzed with a 95% confidence interval using MINITAB 24 software (Version 21.4.3).

#### Regression analysis

Regression analysis is a statistical method used to model the relationship between a dependent variable (*y*) and one or more independent variables (*x*_*1*_, *x*_*2*_, *…*,* x*_*n*_)^79–81^. The main goal is to estimate the coefficients of the regression equation, explaining the relationship between the variables^82^. Additionally, normal probability plot (NPP) was included to assess the residuals from model. This is crucial in regression analysis, as normality of residuals is a key assumption for the validity of the model.

Regression Equation for thermal conductivity and flexural strength depicted in Eqs. (7) and (8)

7 $$\:{K}_{\text{cond}}={a}_{0}+{a}_{1}\left(\text{Gypsum}\right)+{a}_{2}\left(\text{P}\text{M}\text{S}\text{C}\right)+{a}_{3}\left(\text{Thickness}\right)$$

8 $$\:{\varvec{\sigma}}_{\text{flex}}={\varvec{b}}_{0}+{\varvec{b}}_{1}\left(\text{Gypsum}\right)+{\varvec{b}}_{2}\left(\text{PMSC}\right)+{\varvec{b}}_{3}\left(\text{Thickness}\right)$$

where, $\:{K}_{\text{cond}}$Thermal conductivity of the false ceiling tile (W/mK). ${\sigma}_{\text{flex}}$Flexural strength of the false ceiling tile (MPa). Gypsum: Percentage of gypsum used in the composite (% by weight). PMSC: Percentage of Pearl Millet Seed Coverings in the composite (% by weight). Thickness: Tile thickness (mm). $\:{a}_{0},\:{a}_{1},\:{a}_{2},\:{a}_{3,\:}{\:b}_{0},{\:b}_{1},\:{b}_{2},\:{b}_{3}$ Regression coefficients for thermal conductivity and flexural strength respectively.

## Results and discussion

Three samples were prepared and tested for each composition to ensure the reliability of the experimental results. This approach allowed for the assessment of variability within each test condition and contributed to the statistical robustness of the findings.

### Experimental results

**Fig. 5:**
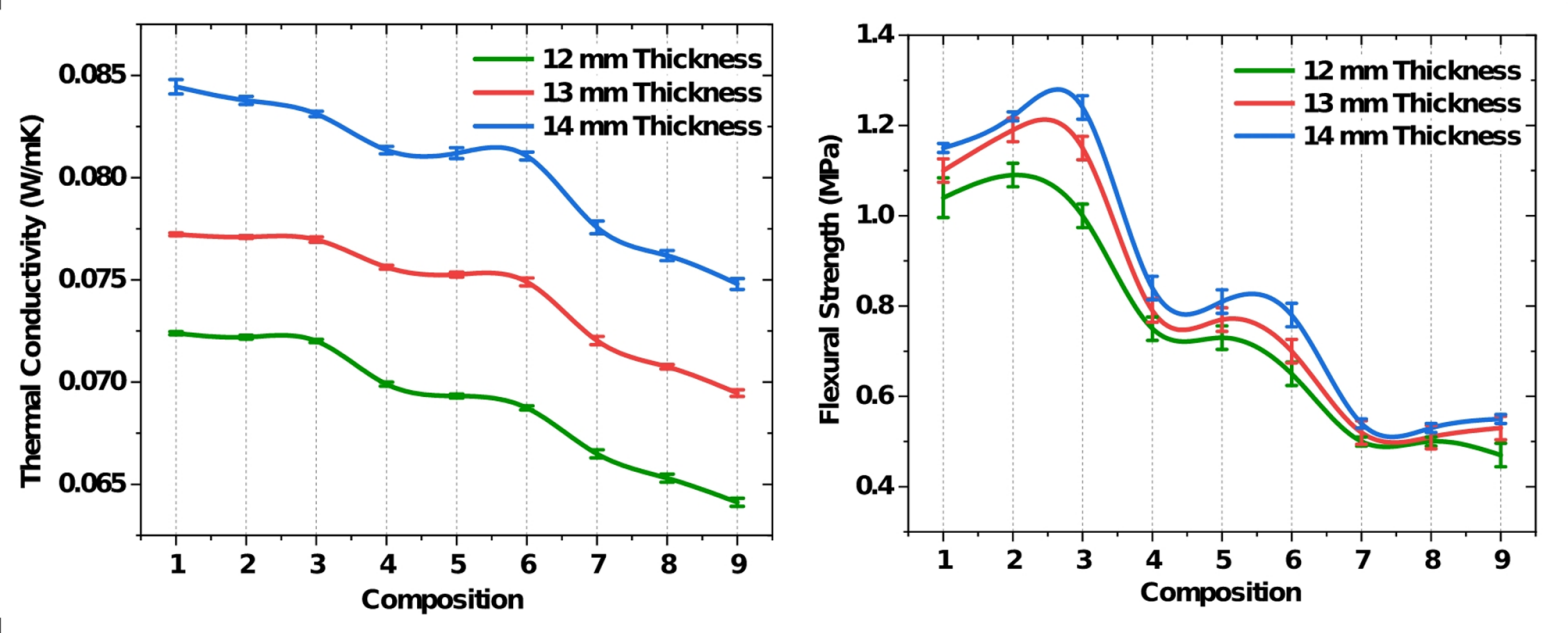
Thermal conductivity and flexural strength.

The experiments were conducted on the biocomposite ceiling tiles to determine thermal conductivity and flexural strength. The analyzed results, presented in Fig. 5, reveals that the thermal conductivity varied across the tested compositions. The minimum thermal conductivity value of 0.0652 W/mK was observed in a composition containing 10% gypsum and 45% pearl millet waste at a thickness of 12 mm. This value is comparable to that of gypsum ceiling tile, which has a thermal conductivity of 0.0598 W/mK. This indicates that reducing the gypsum while increasing the proportion of waste significantly enhances the thermal insulation properties of the ceiling tile. It aligns closely with results for groundnut shell and coir composites (0.066 W/mK)^29^ and wheat straw and corn husk (0.047 W/mK)^28^. These findings align with the insulating nature of natural fibers, which contribute to lower thermal conductivity.

The maximum flexural strength of 1.24 MPa was achieved with a composition comprising 30% gypsum and 55% pearl millet waste for 14 mm of thickness. The value is closely comparable to commercially available gypsum ceiling tile, which have a flexural strength of 1.33 MPa. The result indicates that increasing the proportion of waste and gypsum positively affects the mechanical strength of the tiles, with a thicker composition enhancing structural integrity. However, excessive gypsum reduction negatively affected flexural strength, suggesting the need for a balanced composition to achieve mechanical robustness. It surpasses the results reported for composites reinforced with rice husk and vine pruning (0.08–0.28 MPa)^30^ and those with wheat straw and corn husk (0.80 MPa)^28^. While higher flexural strength values were reported in studies using materials such as groundnut shell and coir, up to 3.6 MPa^29^.

The standard deviation reveals the consistency experimental outcomes. Among the three thicknesses, 12 mm tiles exhibited relatively stable thermal behavior with lower deviations. As tile thickness increased, a gradual rise in variability was observed. This suggests that thicker tiles may be more susceptible to uneven distribution during mixing and moulding processes. The standard deviation analysis of flexural strength indicates that tiles with 12 mm and 13 mm thicknesses exhibited lower variability in flexural performance, indicating a more uniform load distribution and better bonding among the constituents. In contrast, the 14 mm thick tiles showed higher standard deviation values across multiple compositions, suggesting greater deviation in mechanical properties. This could be attributed to internal structural imperfections or uneven stress distribution during testing, which are more pronounced in thicker samples.

### Signal-to-Noise (S/N) ratio analysis

**Fig. 6:**
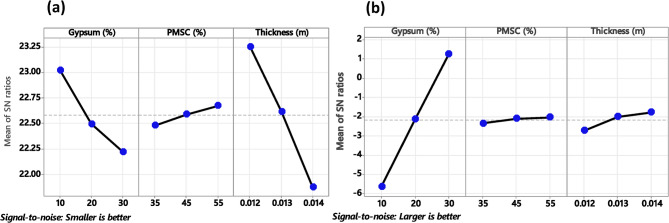
Main effect Plot for SN ratio (**a**) Thermal conductivity (**b**) Flexural strength.

The results of the S/N ratio analysis for thermal conductivity and flexural strength are summarized in the response Table 5 and main effect plot, Fig. 6a,b. The main effects plot shows how different levels of a parameter affect the response characteristic, such as the S/N ratio. A main effect exists when varying levels of a parameter, results in different response characteristics. The slope of the lines in the main effects plot indicates the relative magnitude of the parameter effects. A steeper slope suggests a more significant effect on the response variable^83,84^. Among selected parameter ceiling tile thickness is identified as the most influential parameter for thermal conductivity, with the highest delta value (1.38) and the highest rank, followed by gypsum (delta = 0.80) and PMSC (delta = 0.20). For thickness, the smallest level (12 mm) results in the highest S/N ratio, suggesting that reducing the thickness minimizes thermal conductivity. Similarly, for gypsum (10%) and pearl millet waste (55%), the levels corresponding to the highest S/N ratios are optimal for achieving better thermal performance.

**Table 5:** Signal to noise Ratios.

Level	S/*N* ratio (thermal conductivity)			Level	S/*N* ratio (flexural strength)		
	Gypsum	PMSC	Thickness		Gypsum	PMSC	Thickness
1	23.02	22.48	23.25	1	−5.629	−2.353	−2.726
2	22.50	22.59	22.62	2	−2.126	−2.113	−2.017
3	22.22	22.68	21.87	3	1.240	−2.048	−1.771
Delta	0.80	0.20	1.38	Delta	6.869	0.305	0.955
Rank	2	3	1	Rank	1	3	2

The Larger-the-Better S/N ratio was applied for flexural strength to maximize the ability material to resist bending stresses. The S/N ratio reveals that gypsum is the most influential parameter, with the highest delta value (6.869) and the highest rank. This is followed by thickness (delta = 0.955, rank 2) and PMSC (delta = 0.305, rank 3). The main effect plot highlights that for gypsum, the third level (30%) yields the highest S/N ratio, indicating optimal flexural strength performance. For thickness, the highest S/N ratio is observed at the largest level (14 mm), while PMSC shows minimal variation across levels, indicating a relatively lesser impact on flexural strength.

While the results clearly identify the dominant factors, a deeper understanding of their influence enhances the interpretation. Thinner ceiling tiles reduce the overall distance for heat transfer, limiting thermal conduction paths and thereby lowering thermal conductivity. Moreover, the compact structure in thinner tiles may minimize air gaps, further enhancing insulation. On the other hand, role of gypsum in flexural strength attributed to its function as a continuous matrix phase, which reinforces structural rigidity and improves the ability of material to resist bending stresses. In contrast, pearl millet waste serves primarily as a thermal insulator with lower mechanical contribution. This distinction clarifies that the thickness governs thermal performance, while gypsum significantly affects mechanical strength.

### Interaction plot analysis

**Fig. 7:**
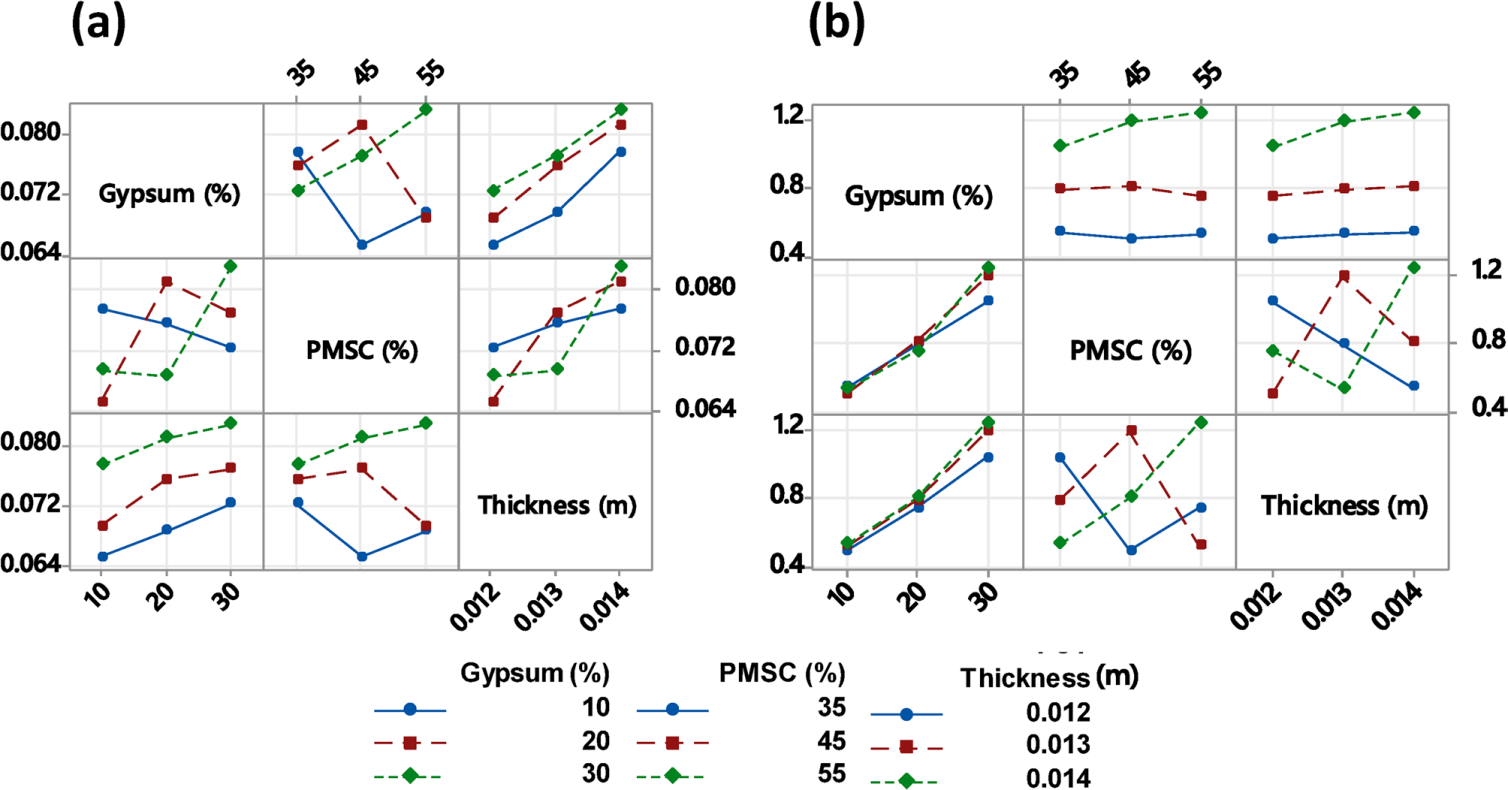
Interaction plot (**a**) Thermal conductivity (**b**) Flexural strength.

The main effects plot shows how different levels of a parameter affect the response (thermal conductivity and flexural strength) characteristic, such as the S/N ratio. The interaction plots were generated to examine the effect of one parameter on the level of another parameter. The association between parameters can be analyzed based on the orientation of the lines. Parallel lines indicate no association between parameters, while non-parallel lines suggest the presence of interaction effects. The strongest association are observed when lines are nearly perpendicular^85,86^. In Fig. 7a the subplots between pearl millet waste and gypsum signifying strong association. In most cases, higher waste content with gypsum corresponds to an increase in thermal conductivity. However, the degree of increase is dependent on both gypsum and thickness. The trend shows that as gypsum increases from 10 to 30%, the thermal conductivity varies in a non-linear manner, indicating gypsum influences the thermal properties depending on the waste and thickness. The results presented in Fig. 7b highlight that flexural strength is determined by complex interplay of material composition (waste and gypsum) and thickness, rather than single parameter. The most pronounced association appear between waste and thickness. Additionally, the interaction between gypsum and waste is evident, as the flexural strength varies non-linearly when changing the PMSC and gypsum content.

### Analysis of variance (ANOVA)

For thermal conductivity (Table 6), the regression model demonstrated a high coefficient of determination (R^2^ = 98.88%), indicating that the model effectively explains the variability in the data. The adjusted R^2^ (98.21%) and predicted R^2^ (96.90%) further validate the robustness of the model. Among the individual parameters, gypsum and tiles thickness exhibited significant effects on thermal conductivity, with F-values of 107.87 and 330.18, respectively, and corresponding P-values < 0.05, signifying their strong influence. In contrast, PMSC showed a marginal effect (P-value = 0.08), suggesting limited contribution to thermal conductivity under the given experimental conditions.

For flexural strength (Table 7), the regression model revealed a high level of accuracy, with R^2^adjusted R^2^and predicted R^2^ values of 98.09%, 96.95%, and 94.44%, respectively. Gypsum emerged as the most influential factor, with an F-value of 249.19 and a P-value < 0.05, underscoring its dominant role in determining flexural strength. The tile thickness was significantly affected flexural strength (F-value = 61.25, P-value = 0.05). However, the effect of PMSC was insignificant (P-value = 0.26), suggesting that it lacks in contribute to enhance the flexural properties.

**Table 6:** ANOVA model summary for thermal conductivity.

Source	DF	Adj SS	Adj MS	F-Value	*P*-Value
Regression	3	0.000281	0.000094	147.61	0.00
Gypsum	1	0.000068	0.000068	107.87	0.00
PMSC	1	0.000003	0.000003	4.78	0.08
Thickness	1	0.000210	0.000210	330.18	0.00
Error	5	0.000003	0.000001		
Total	8	0.000284			
**S**	**R-sq**		**R-sq (adj)**		**R-sq (pred)**
0.0007968	98.88%		98.21%		96.90%

**Table 7:** ANOVA model summary for flexural strength.

Source	DF	Adj SS	Adj MS	F-Value	*P*-Value
Regression	3	0.620417	0.206806	85.65	0.00
Gypsum	1	0.601667	0.601667	249.19	0.00
PMSC	1	0.003750	0.003750	1.55	0.26
Thickness	1	0.015000	0.015000	6.21	0.05
Error	5	0.012072	0.002414		
Total	8	0.632489			
**S**	**R-sq**		**R-sq (adj)**		**R-sq (pred)**
0.0491370	98.09%		96.95%		94.44%

Although pearl millet waste constitutes a significant proportion of the biocomposite formulation, its statistical influence on thermal conductivity (*P* = 0.08) and flexural strength (*P* = 0.26) was found to be marginal. This can be attributed to its secondary role as a fibrous reinforcement. waste contributes to thermal insulation through its inherent low thermal conductivity, but this effect becomes prominent in interaction with other parameters, particularly gypsum and tile thickness. In terms of load-bearing capacity of the biocomposite under flexural strength, gypsum forms a rigid and cohesive matrix capable of distributing applied loads across the tile while waste acts as reinforcement tends to poor bonding. As a result, under flexural loading, the biocomposite weakens at the matrix-fiber interface, thereby falling potential of waste to enhance strength. Thus, despite its sustainability value and role in reducing material density, influence of waste on flexural performance remains marginal when compared to the dominant structural contribution of gypsum.

### Regression analysis

Regression analysis was conducted to model the relationships between the independent variables (gypsum, PMSC and thickness) and the dependent performance metrics such as thermal conductivity and flexural strength. The analysis provided predictive Eqs. (9) and (10) and quantified the influence of each parameter. Thickness had the most significant influence for both thermal conductivity and flexural strength. Additionally, Gypsum emerge as strong contributors to flexural strength with minor but positive impact of PMSC, suggesting its potential as a secondary reinforcing material. Similarly, PMSC incorporation contributes to a minor reduction in thermal conductivity.

9 $$\:{K}_{\text{cond}}=-0.00590\:+\:0.000338\:Gypsum-\:0.000071\:PMSC+5.911\:Thicknes$$

10 $$\:{\sigma\:}_{\text{flex}}=-0.575\:+\:0.03167\:Gypsum+\:0.00250\:PMSC+50.0\:Thicknes\:$$

where $\:{K}_{\text{cond}}$ thermal conductivity of the false ceiling tile (W/mK). $\:{\varvec{\sigma\:}}_{\text{flex}}$ flexural strength of the false ceiling tile (MPa). Gypsum: Percentage of gypsum used in the composite (% by weight). PMSC: Percentage of Pearl Millet Seed Coverings in the composite (% by weight). Thickness: Tile thickness (mm).

**Fig. 8:**
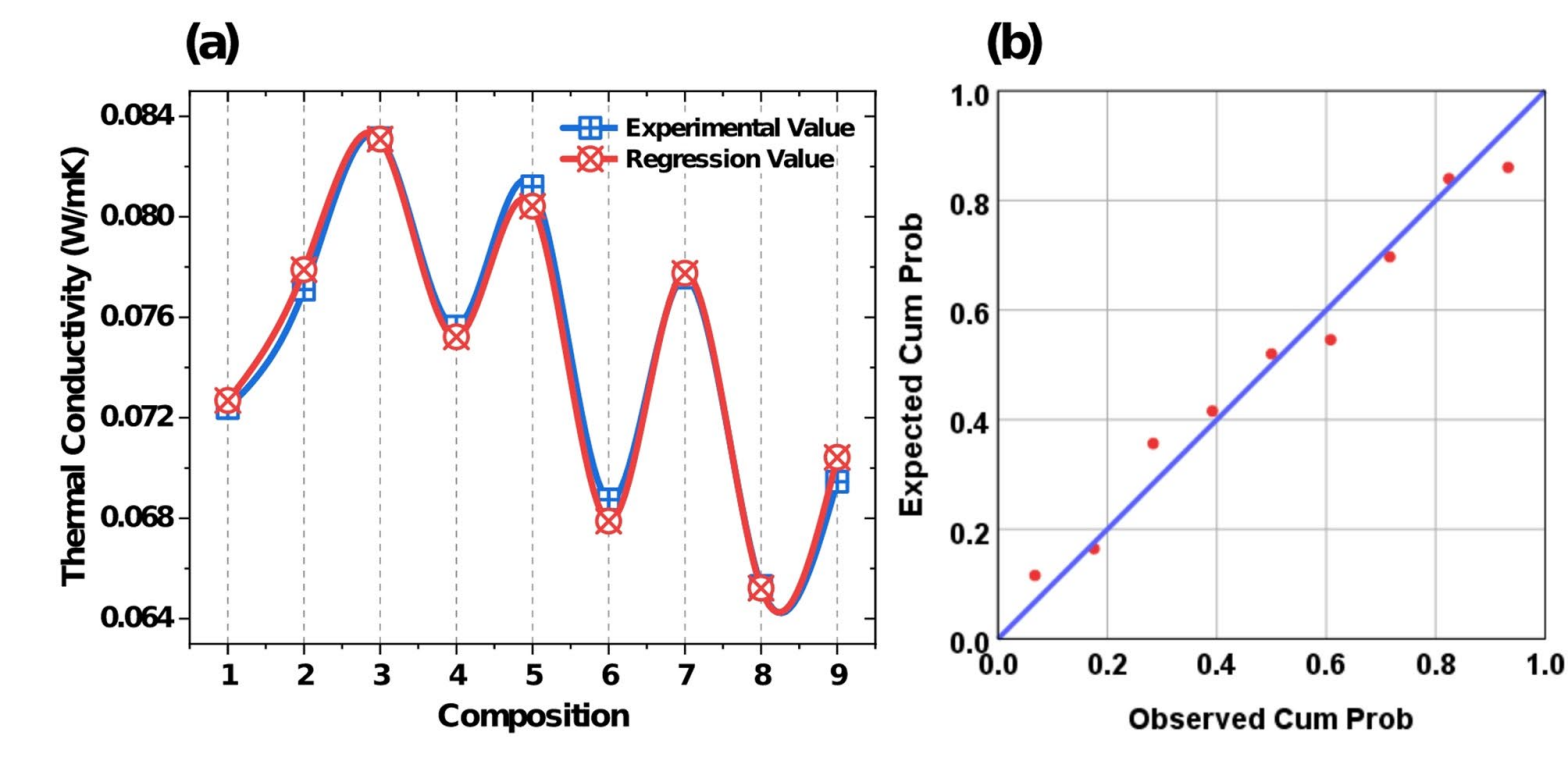
(**a**) Comparison of experimental and regression values of thermal conductivity (**b**) Normal probability plot of residuals.

The comparison between experimental and regression values for thermal conductivity is depicted in Fig. 8a. The trend demonstrate a high degree of agreement between the experimental values and regression values across the nine compositions. The predicted values closely follow the experimental trend, with minimal deviations, indicating the reliability of regression model. The normal probability plots of regression standardized residuals, shown in Fig. 8b for thermal conductivity demonstrate the goodness-of-fit of the regression models.

**Fig. 9:**
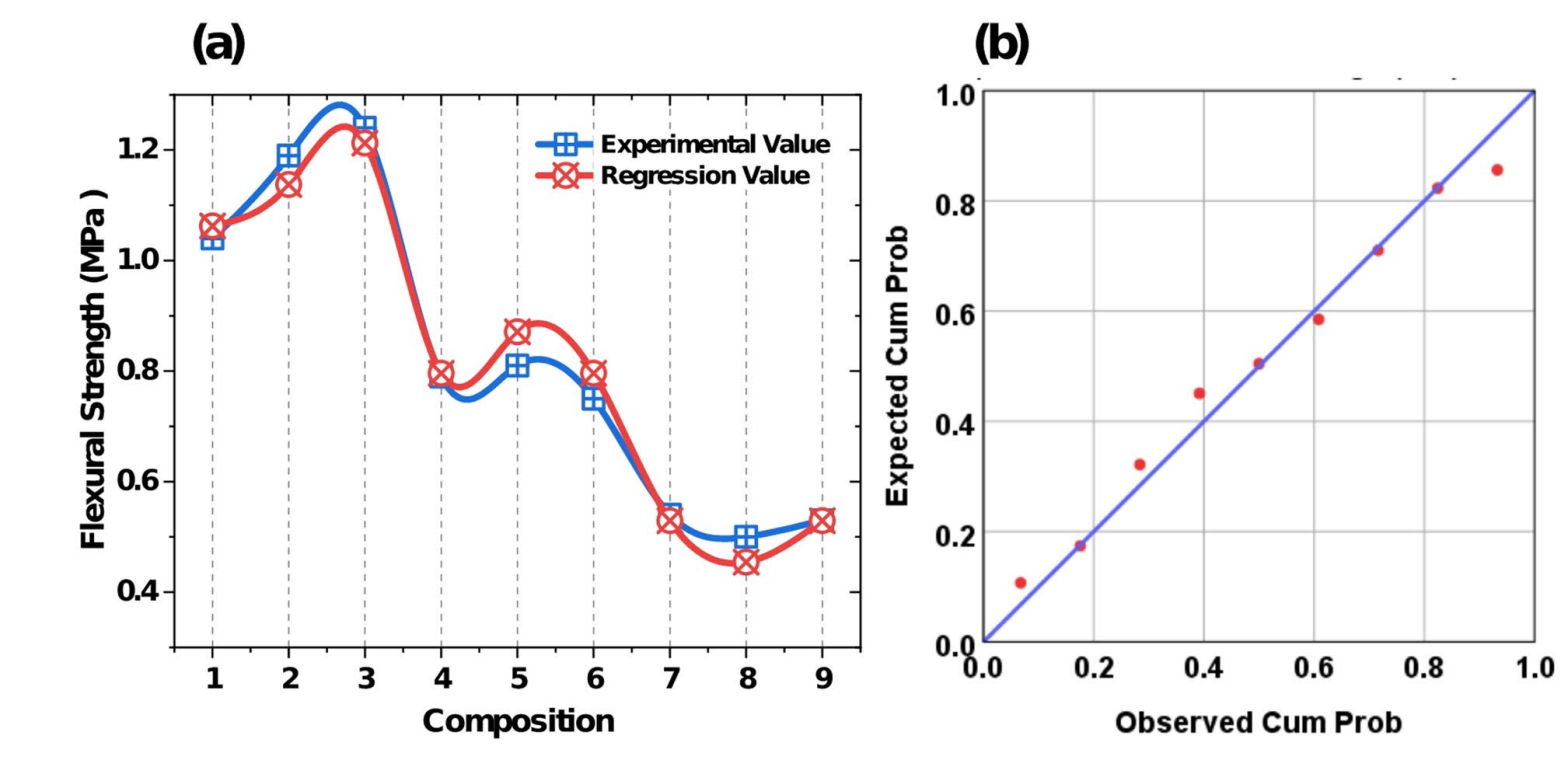
(**a**) Comparison of experimental and regression values of flexural strength (**b**) Normal probability plot of residuals.

The regression values of flexural strength align well with the experimental results, shown in Fig. 9a. It reflecting the ability of model to accurately predict the output responses. The minor discrepancies observed can be attributed to experimental unaccounted variability. The normal probability plots of regression standardized residuals, shown in Fig. 9b for flexural strength demonstrate the goodness-of-fit of the regression model.

**Fig. 10:**
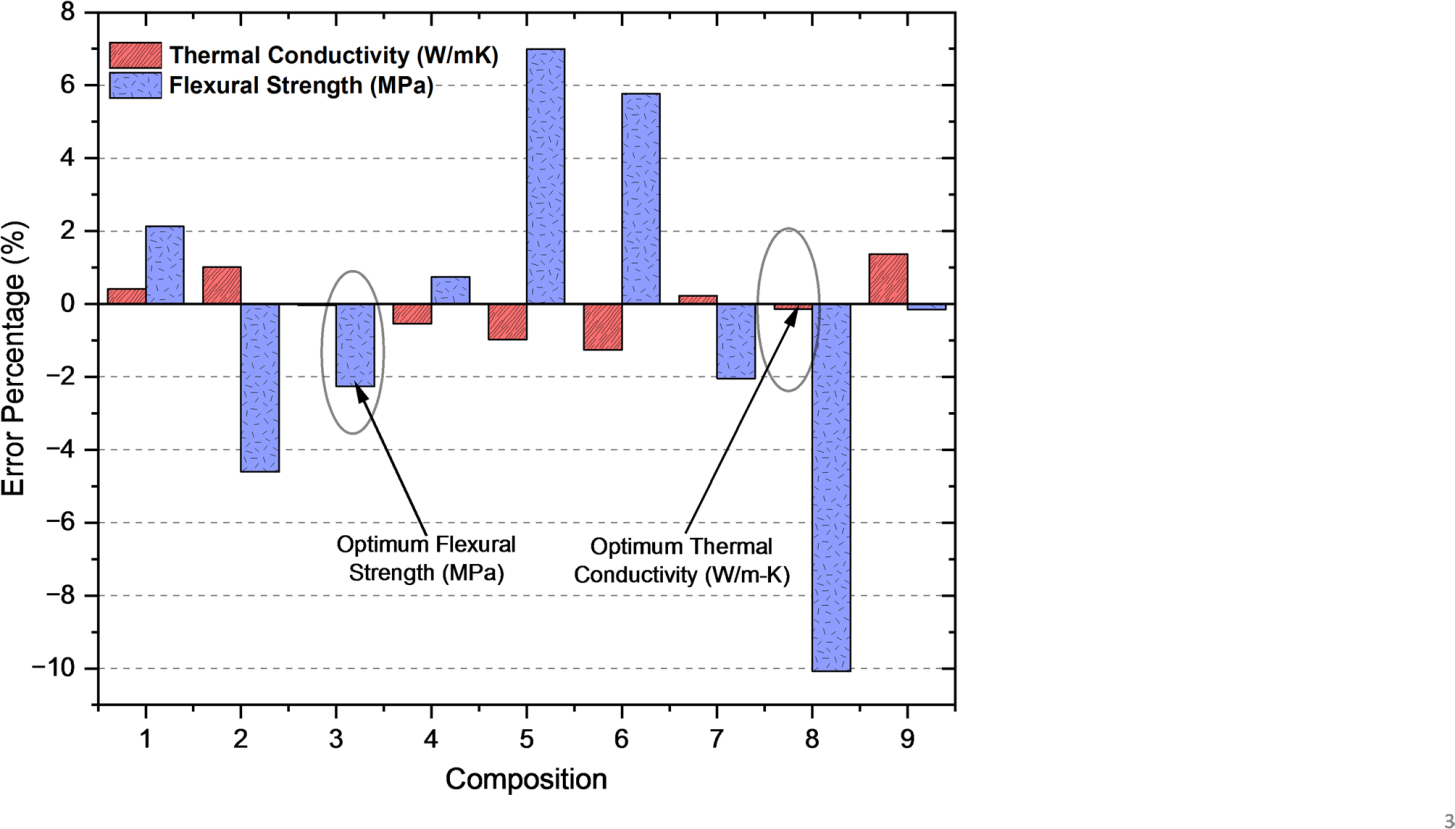
Error percentage in thermal conductivity and flexural strength.

Figure 10 illustrates the error percentages between the experimental and regression-predicted values for thermal conductivity (W/mK) and flexural strength (MPa) across nine experiments. The error percentages for most experiments remain within acceptable limits, demonstrating the robustness and accuracy of the regression model in predicting these properties. The optimal parameter combinations for these properties are highlighted within the plot. In set of experiment 3, the optimum parameter combination for flexural strength achieves a low error percentage of −2.26%, indicating significant predictive accuracy. Similarly, in set of experiment 8, the optimal parameter combination for thermal conductivity yields a minimal error percentage of −0.13%, further validating the reliability of the regression model in accurately predicting these properties.

The determination of optimal parameter combinations ensures the desired performance characteristics of the developed biocomposites. Based on the Signal-to-Noise (S/N) ratio and statistical evaluations, the optimal levels of each factor were identified (Table 8) to achieve the best outcomes for both thermal conductivity and flexural strength. For thermal conductivity, the optimal combination was found to consist of 10% gypsum, 45% PMSC and thickness of 12 mm, resulting in a thermal conductivity value of 0.0653 W/mK. This combination minimizes heat transfer and aligns with the objective of enhanced thermal insulation properties. In contrast, for flexural strength, the optimal combination was identified as 30% gypsum, 55% PMSC and an thickness of 14 mm, yielding a flexural strength of 1.24 MPa. This combination ensures maximum material strength, making the tiles suitable for structural applications.

**Table 8:** Optimum set of response.

Gypsum (%)	PMSC (%)	Thickness (mm)	Thermal conductivity (W/mK)
10	45	12	0.0653
Gypsum (%)	PMSC (%)	Thickness (mm)	Flexural Strength (MPa)
30	55	14	1.24

### Contour plot

**Fig. 11:**
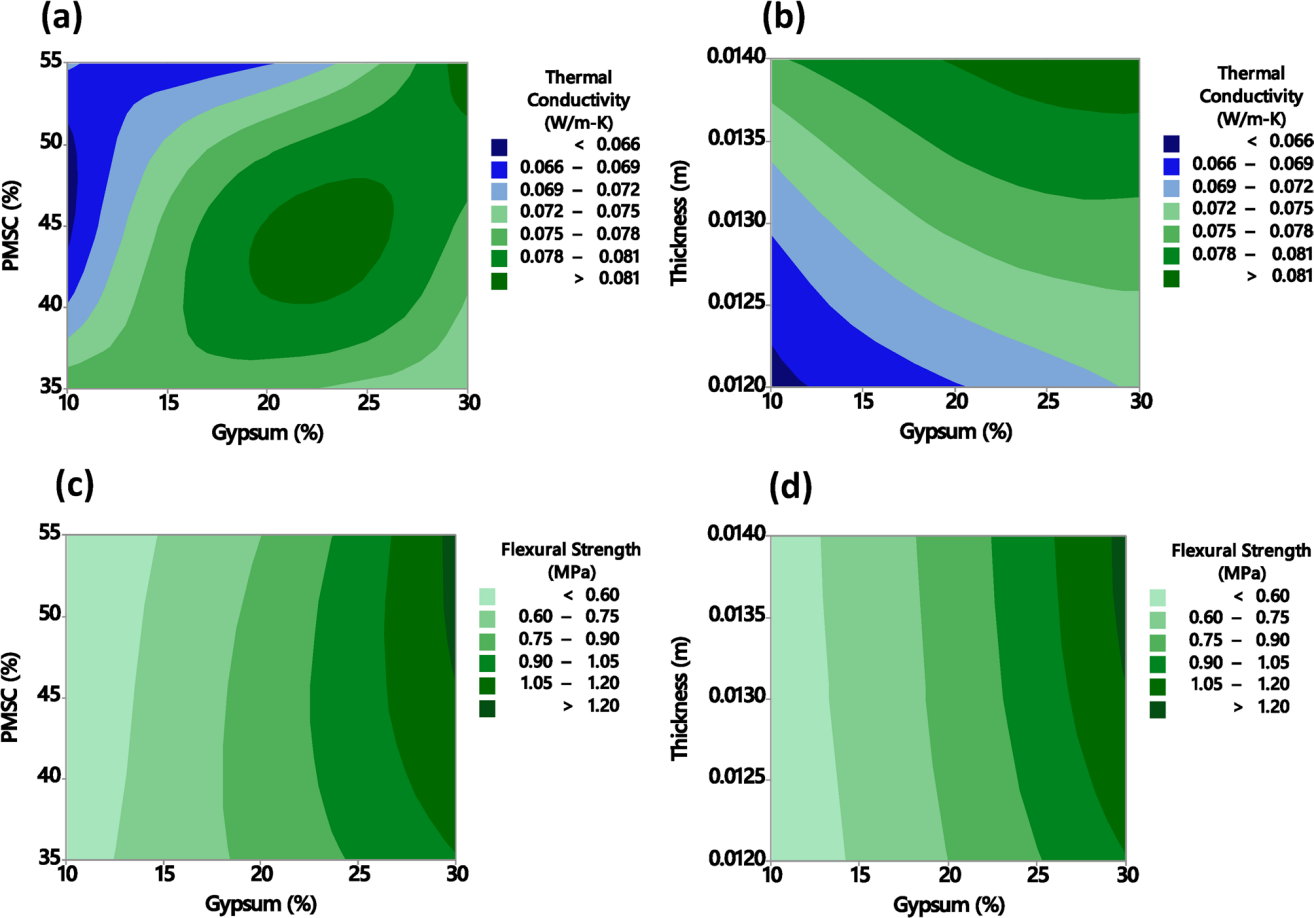
Contour plots showing the effect of gypsum, PMSC and tile thickness on thermal conductivity and flexural strength.

The Contour Plot serves as a visual tool to complement the statistical findings by mapping the combined effects of two input parameters on the response variable in a two-dimensional space^87^. This enhances the interpretation of association identified earlier and aids in identifying optimal process conditions. The contour plots presented in Fig. 11 illustrate the influence of varying pearl millet waste, gypsum and thickness on the thermal conductivity and flexural strength. In Fig. 11a, the combination of higher waste (around 50–55%) and moderate gypsum (15–20%) is associated with the lowest thermal conductivity values (< 0.065 W/mK), highlighting the synergistic effect of these components in enhancing insulation properties. Similarly, Fig. 11b shows that reducing thickness below 0.013 m further decreases thermal conductivity, reflecting improved energy efficiency in thinner tiles. Figure 11c indicates that optimal mechanical performance (> 1.2 MPa) is achieved at higher waste levels (50–55%) and higher gypsum levels. Furthermore, Fig. 11d reveals a positive correlation between thickness and flexural strength, with thicker tiles (> 0.013 m) exhibiting more mechanical robustness. These results collectively highlight that the thermal and mechanical properties can be fine-tuned by adjusting the proportions of waste, gypsum, and thickness.

Enhancement in biocomposite performance at specific gypsum levels can be attributed to the hydration reaction of gypsum during setting^88^. At moderate gypsum levels (20-30%), the matrix is sufficiently continuous to ensure strong interfacial bonding with the embedded waste and other components, enabling effective stress transfer under flexural loads. Simultaneously, the partially porous nature of the hydrated gypsum network allows for moderate insulation by entrapping air pockets. However, excessively high or low gypsum disrupts this balance. Higher gypsum increases thermal conductivity due to its higher intrinsic conductivity, while lower compromises the structural cohesiveness and binding strength. Thus, an optimal range of gypsum supports structural performance through strong bonding and uniform matrix formation along with controls heat transfer characteristics, contributing to the overall functionality of the ceiling tile.

The performance of the developed biocomposite ceiling tiles demonstrates potential when compared to existing studies on pearl millet waste-based composites. Using millet waste in formation of laterite-based bricks significantly reduced thermal conductivity, from 1.4 W/mK for dry pure laterite blocks to 0.29 W/mK, while dropping mechanical strength, as per the increased porosity and lower material density^36,37^. The finding aligns with the present study, where the integration of waste reduces the thermal conductivity of biocomposite. The comparative statement provides insight of waste used in construction materials validating its performance as sustainable alternative.

## Conclusions

This study demonstrates the development and optimization of ceiling tiles by partially replacing gypsum with pearl millet waste. The integration of PMSC as an agricultural waste, coupled with gypsum, waste paper and WWF, presents a novel approach addressing the environmental challenges associated with gypsum extraction, waste burning and energy efficiency in building materials. The optimization process, employing the Taguchi DOE, systematically analyzed the effects of gypsum, waste proportion, and tile thickness on the thermal and mechanical properties. These tiles offer practical applications in ceilings, partition walls, and decorative finishes across diverse geographic regions, making a meaningful contribution to environmental sustainability and the advancement of energy-efficient building solutions.

The findings underscore the significance of these parameters in achieving optimal performance. The present study finds experimental results highlights minimum thermal conductivity of 0.065 W/mK, comparable to gypsum tiles founds at 10% gypsum, 45% waste, and 12 mm thickness. For flexural strength, the ideal composition was 30% gypsum, 55% waste, and 14 mm thickness, reaching 1.24 MPa. The S/N ratio analysis revealed the relative influence of each parameter. Thickness emerged as the most significant factor for thermal conductivity (Δ = 1.38), due to its direct effect on heat transfer pathways. Gypsum content, with a delta value of 0.80, also played a substantial role due to its higher conductivity. In contrast, PMSC had a minimal effect (Δ = 0.20), due to its contribution to insulation. For flexural strength, gypsum content dominated with the highest delta (Δ = 6.869), confirming its role in matrix formation and mechanical load bearing. Thickness followed (Δ = 0.955), while PMSC had the least influence (Δ = 0.305), consistent with its limited bonding capacity and lack of structural stiffness. ANOVA results supported these conclusions statistically. For thermal conductivity, gypsum and thickness had statistically significant effects (*P* = 0.00), however PMSC had a P-value of 0.08, indicating marginal impact. Similarly, for flexural strength, gypsum (*P* = 0.00) and thickness (*P* = 0.05) were statistically significant, while PMSC (*P* = 0.26) was not. These findings suggest that while pearl millet waste contributes to sustainability and thermal insulation, it functions more as a passive filler than an active strengthener.

The developed regression models attained high accuracy for thermal conductivity and flexural strength, with R^2^ values of 96.90% and 94.44%, respectively. The low error percentages (< 3%) between experimental and predicted values validated the reliability of the models. Additionally, interaction and contour plots revealed that optimal performance is achieved through a balanced mix. With moderate gypsum content for matrix integrity, higher waste for insulation and controlled thickness to tune thermal and mechanical properties. A strong association between waste and gypsum was observed in interaction plot for thermal conductivity. Similarly, the strongest interaction for flexural strength was observed between waste content and thickness, indicating their significant combined influence. The contour plots demonstrate an optimal blend of 50–55% waste and 15–20% gypsum significantly reduces thermal conductivity (< 0.065 W/mK), enhancing insulation. Additionally, thinner tiles (< 0.013 m) improve energy efficiency. For mechanical performance, higher waste and gypsum content (> 1.2 MPa flexural strength) strengthen the tiles, while greater thickness (> 0.013 m) enhances structural integrity.

### Research contributions and future scope

Beyond material performance, this study offers substantial practical and environmental benefits. pearl millet waste, being an abundant agricultural by-product, is readily available at low or no cost, making the production of ceiling tile biocomposite more economical. By reducing gypsum usage, the process also lowers environmental impact associated with mining, processing and landfill waste. Utilisation of biodegradable components such as pearl millet waste, WWF and waste paper pulp further enhances recyclability at the end phase. Additionally, the fabrication process relies on low-energy mechanical mixing and ambient drying, makes it feasible for small-scale or decentralized production in rural or resource-limited settings.

This study provides an original contribution to sustainable construction materials by introducing PMSC as a key component in partially replacing gypsum in ceiling tile. The findings serve as a foundation for future research on agricultural waste-based biocomposites, with potential applications in various sectors of the construction industry. Further studies can explore the cost analysis, ageing studies, Life Cycle Assessment (LCA), acoustic properties, and fire resistance of biocomposite ceiling tile to broaden their applicability. Additionally, scaling up to mass-production and assessing recyclability of these tiles in real-world applications are areas for future investigation. Conducting a LCA will provide insights into the environmental impact across the lifespan of the tile. In addition to material performance evaluation, future studies should also examine the scalability and manufacturing feasibility of the developed biocomposite ceiling tiles. Investigating the potential for large-scale production using low-cost, readily available technologies is essential to determine their suitability for commercial deployment.

## Data Availability

All data generated or analysed during this study are included in this article.
